# Social hierarchy modulates responses of fish exposed to contaminants of emerging concern

**DOI:** 10.1371/journal.pone.0186807

**Published:** 2017-10-19

**Authors:** Jelena Ivanova, Shiju Zhang, Rong-Lin Wang, Heiko L. Schoenfuss

**Affiliations:** 1 Aquatic Toxicology Laboratory, Saint Cloud State University, Saint Cloud, Minnesota, United States of America; 2 Department of Mathematics & Statistics, Saint Cloud State University, Saint Cloud, Minnesota, United States of America; 3 Exposure Methods & Measurements Division, National Exposure Research Laboratory, US Environmental Protection Agency, Cincinnati, Ohio, United States of America; Universite de Liege, BELGIUM

## Abstract

Many organisms, including the fathead minnow (*Pimephales promelas*), a toxicological model organism, establish social hierarchies. The social rank of each male in a population is under the control of the hypothalamic-pituitary-gonadal (HPG) axis mainly through regulation of circulating androgen concentrations, which in turn drive the expression of secondary sex characteristics (SSCs). As dominant and subordinate males in an exposure study are initially under different physiological conditions (i.e., differing plasma androgen concentrations), we proposed that they belong to different subpopulations in the context of exposure to compounds that may interact with the HPG axis. Using a meta-analysis of our data from several previously published studies, we corroborated the hypothesis that social status, as indicated by SSCs, results in distinct clusters (eigenvalues >0.8 explaining >80% of variability) with differential expression of plasma vitellogenin, a commonly used biomarker of exposure to contaminants of emerging concern (CEC). Furthermore, we confirmed our predictions that exposure to estrogenic CECs would homogenize plasma vitellogenin response (E1: cluster mean SSC values decreased to 4.33 and 4.86 relative to those of control; E2: decreased to 4.8 and 5.37) across the social hierarchy. In contrast, serotonin-specific reuptake inhibitors expand this response range (cluster mean SSC increased to 5.21 and 6.5 relative to those of control). Our results demonstrated that social hierarchies in male fathead minnows result in heterogeneous responses to chemical exposure. These results represent a cautionary note for the experimental design of single-sex exposure studies. We anticipate our study to be a starting point for the re-evaluation of toxicological data analyses in single sex exposure experiments.

## Introduction

Life science research frequently relies on the use of laboratory organisms (e.g., bacteria, plants, or animals) to test experimental hypotheses. The use of these organisms in laboratory cultures and in experimental testing requires a high degree of homogeneity to minimize experimental variance due to random error. Indeed, the common practice of providing sample sizes per treatment in the methods section of scientific publications implicitly assumes that replicate test subjects are similar enough to allow treatment effects of interest to be detected at a desired level of statistical confidence. This assumption, however, runs counter to the principles of evolutionary biology with regard to individual variability (unless parthogenetic organisms such as *Daphnia magna* are used for toxicological experiments). Toxicology, which frequently attempts to extrapolate from effects observed in individual organisms to consequences affecting populations of conspecifics, seldom takes into account the impact of manifested genetic/epigenetic/organismal variation in a population (see [1]). The objective of the current study was to investigate the presence of social hierarchies in single sex fish populations used in toxicological studies and determine how social status modulates the expression of biomarkers following exposure to CECs.

While intraspecific variability may arise as a result of many different biological processes, dominant/subordinate relationships among individuals of the same species are regarded as important contributing factors [2,3]. When a social hierarchy becomes established, the ranking of each individual is frequently based on outcomes of aggressive encounters [2]. In sexually dimorphic species, the social ranks of males are often associated with the expression of secondary sex characteristics (SSCs), and may be indicative of reproductive condition [4]. Social hierarchies are dynamic and subject to change. For example, when a dominant male loses its advantage in a population, it may be replaced by a subordinate male. This leads to physiological transformations, enhanced SSCs, and greater fitness in the latter [5]. Social hierarchy can develop under both natural and laboratory conditions [6], and may have implications for toxicological studies. Indeed, there is evidence suggesting that the endocrine physiology of an animal could be modulated by its social status [2,7], and that social status is sensitive to exposure to contaminants of emerging concern (CECs). Such contaminants have become ubiquitous in anthropogenically-altered environments [8].

**Fig 1:**
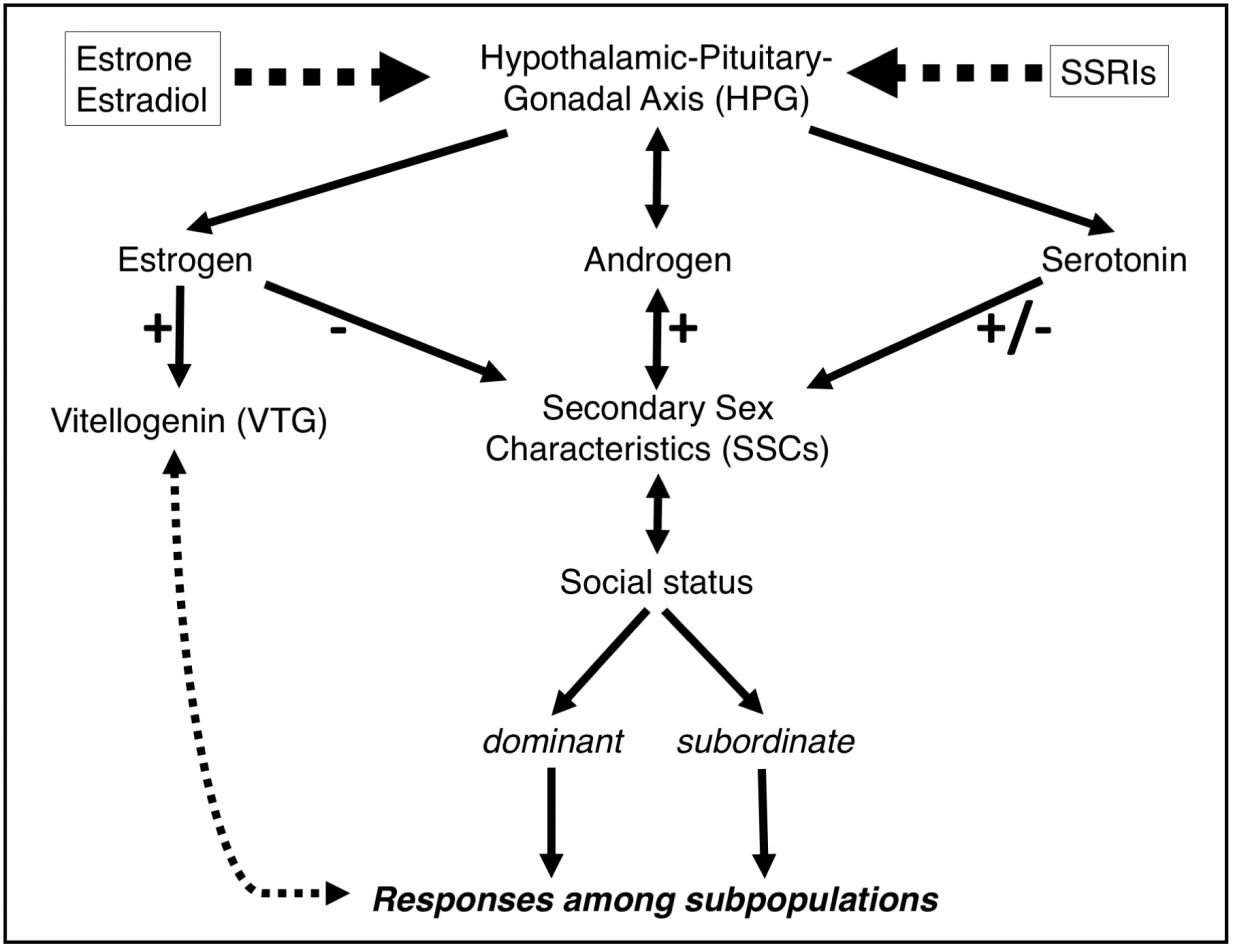
Conceptual framework for the current study. Conceptual drawing of HPG axis and the impact of modulators (Estrone E1, Estradiol E2, and Serotonin-selective reuptake inhibitors SSRIs) on social status as a result of changes in secondary sex characteristics. We hypothesize that dominant subpopulations will respond differently to an estrogenic contaminant than subordinate subpopulations. In the current study, vitellogenin biosynthesis in male fathead minnows was used to assess the estrogenic exposure effect in the two subpopulations. Plus (+) and minus (-) symbols indicate stimulatory or inhibitory effects, respectively.

Multiple pathways interact in intricate modulation of the endocrine system (Fig 1), with the brain integrating external and internal stimuli to establish an appropriate endocrine response for each individual [6]. The hypothalamic-pituitary-gonadal (HPG) axis regulates the production of sex hormones, which in turn guide sexual maturation and reproductive success [9]. In contrast, the hypothalamic-pituitary-adrenal (HPA) axis responds to external and internal stressors, often through the release of the cortisol hormone. Differing levels of stress are imposed upon an animal based in part on its social status, especially on the subordinate individuals [5,7]. In addition, recent studies suggest that neurological circuits in the central nervous system (S1 Fig) might be altered as a result of interactions between dominant and subordinate conspecifics [10].

Among the most widely studied CECs are the naturally occurring estrogens17 β-estradiol (E2) and its less potent metabolite estrone (E1) [11–16]. Estrogenic hormones have multi-faceted and wide-ranging effects in vertebrates, and are the products of HPG axis activation. Also, well studied are several mood-altering pharmaceuticals acting as selective serotonin reuptake inhibitors (SSRIs) [17–21]. SSRIs are inherently biologically active and often target areas of brain involved in influencing the dominant-subordinate behavior among conspecifics. The subordinate behavior is physiologically determined by a decrease in HPG axis activity and a chronic increase of brain serotonin (5-Hydroxytryptamine, 5-HT) levels [2,22]. The differential effects of serotonin on the brains of animals representing different social statuses presumably reflect their varying responses to SSRIs, which prolong serotonin presence in synaptic clefts. The temporal differences in response to serotonin exposure may widen the gap between the dominants and subordinates and promote the social hierarchy among the male conspecifics. This widening gap may be represented by a greater difference between the SSCs of dominant vs subordinate males.

The molecular pathways involved in the initiation of toxic responses are highly conserved across vertebrates, and the effects of CECs on these pathways have been studied extensively in model laboratory species such as the fathead minnow (*Pimephales promelas*) [9,23]. For example, previous research [24] found high resistance and/or tolerance to pathogens in dominant males and in males with elaborate SSCs [25]. Reproductive success is dependent upon the males’ ability to acquire and defend a high-quality nesting territory in the presence of other male competitors using the prominent expression of SSCs as an indicator of superiority [26]. Thus larger, more dominant individuals may interfere with the general reproductive functions of subordinate fish, particularly in their ability to hold a territory or defend a nest site. In addition, smaller fish require more time to replenish over-winter energy deficits than do larger fish because of their lower energy storage, which results in delayed reproduction [3]. Estrogens are important in oviparous vertebrates because they are involved in a range of biological functions/processes, including the production of vitellogenin (VTG), a yolk precursor protein, in the liver of adult females [27]. However, exposure to exogenous estrogenic compounds can lead to detrimental effects, especially for males. For example, estrogens can induce VTG synthesis in males [11,28], alter SSC expression of adult male fish [9], or lead to incidences of intersex (presence of both male and female gonadal tissues) [29]. As demonstrated by Martinovic et al. [30], male fathead minnows exposed to E2 contained lower levels of circulating androgens, leading to reduced aggressive behavior and impaired ability to acquire a nest site under competitive pressure. Subordinate fish might be more sensitive to CECs as the cells secreting gonadotropin-releasing hormone (GnRH) in subordinate males are eight-fold fewer compared to those of dominant males [5,7].

Researchers have previously noted that the complexity of social structure among vertebrates may have implications in their responses to CECs [17,18,31]. In the mid-1990’s, researchers began to combine studies to explore the behavioral and physiological variabilities due to heterogeneity of animal populations. These trends are often not resolvable in individual studies due to small sample sizes [32], and may require meta-analyses of multiple data sets. For meta-analyses to be successful, however, there must be a significant degree of similarity between studies in order for the pooled analysis to be meaningful. The “quality” of data sets, based on the number and measurement criteria of experimental variables among studies, is often used as a filtering method [33]. This type of evaluation was adopted in the current study by using multivariate statistical analyses to examine the homogeneity of environmental variables of previously published data sets. The filtered data were then analyzed to test the hypothesis that social status, as indicated by the differential display of SSCs, results in altered responses in CEC exposed fish as reflected by differing concentrations of plasma VTG within the same treatment. Furthermore, we predicted that the variability of SSC expression would decrease in male fathead minnow populations exposed to the estrogenic CECs E1 and E2, and would increase upon exposure to SSRIs.

## Materials and methods

### Ethics statement

This study utilized data from previous fish exposure experiments. All studies that contributed data to the current analysis were carried out in strict accordance with the recommendations in the Guide for the Care and Use of Laboratory Animals by the National Institutes of Health. The exposure protocols for all studies included in this meta-analysis were approved by the Institutional Animal Care and Use Committees of St. Cloud State University. Fish were deeply anesthetized (complete loss of response to manual stimulation) in a neutral buffered solution of MS-222 prior to blood collection and were sacrificed by subsequent cervical dislocation. All efforts were made to minimize distress to the animals.

### Study conditions

A meta-analysis integrating multiple previous studies (Table 1; S1 Table) was conducted to explore the heterogeneity of male fathead minnow populations subjected to estrogen or SSRI exposures. All studies were carried out under common exposure conditions and had similarly assessed biological endpoints including three morphometric indices: hepatosomatic index (HSI = liver weight/whole body weight), gonadosomatic index (GSI = gonadal weight/whole body weight), and body condition factor (BCF = (whole body weight/length^3) *100,000).

**Table 1:** Studies contributing data to the current analysis.

study	compounds	measured concentration [ng/L]	# fish/ treatment	water temp [°C± st. dev.]
[16] Dammann (2011)	Control	0	20	21.5±0.3
	17β-Estradiol	2–35	20	
[31] Hyndman (2010) Experiment I and II	Control	0	13	21.8±0.4
	17β-Estradiol	12–17	13	
[34] Kolok (2012) Days 8 and 12	Control	0	15	21.3±0.3
	17β-Estradiol	13.2–36.4	15	
[35] Rearick (2014) *	Control	0	12	21.1±0.4
[36] Schoenfuss (2008)	Control	0	12	24±0.4
[37] Schultz (2012)	Control	0	24	21.1±0.4
	17β-Estradiol	30	24	
[18] Schultz (2011)	Control	0	10	22.9±0.2
	Bupropion	7.4–57	10	
	Venlafaxine	305–1104	10	
	Sertraline	1.6–5.2	10	
	Fluoxetine	2.5–28	10	
[38] Shappell (2010)	Control	0	10	21.6±0.4
	17β-Estradiol	9–44	10	

*nominal concentrations only.

### Exposure

The test organisms in all included studies were 6-month old mature male fathead minnows from the same laboratory fish supplier (Environmental Testing and Consulting laboratory, Superior, WI). Upon arrival at the St. Cloud State University Aquatic Toxicology Laboratory (St. Cloud, MN), all fish were subjected to a 21-day in-house flow-through exposure at constant environmental conditions; including similar photo-period (16:8 light:dark), temperature (20–23°C, S1 Fig), dissolved oxygen (5.0–7.5 mg/L), and pH (7.2–8.3) [16,18,31,34–39]. Exposure experiments followed published flow-through exposure protocols [16]. Briefly, male fathead minnows were randomly assigned to control or exposure aquaria at an approximate density of 10 fish per 16L tank. Fish were maintained according to U.S. Environmental Protection Agency guidelines [40] throughout the experiments, and fed frozen brine shrimp (*Artemia fransiscana*, San Francisco Bay Brand, Inc., Newark, CA) twice daily *ad libitum*.

### Chemicals

Four treatment conditions were examined in the present study: an ethanol carrier control, E1, E2 and SSRIs. The SSRI treatment group contained the data from the exposures to sertraline, fluoxetine and venlafaxine (see Table 1 for additional treatment-specific information). As a condition for the meta-analysis, all concentrations for a treatment condition (for example, E1) were combined. Similarly, all SSRI pharmaceuticals were combined as one SSRI treatment. This approach is commonly applied in meta-analyses, and is justified as the current study attempted to assess whether social status modulates fish responses to a CEC exposure, not the biological effects of the exposure. All data used in this analysis were extracted from the original “raw” data collected at the time of the original studies, and are included in a supplemental file (S1 Table).

### Biological endpoints

At the end of each experiment, a series of endpoints were documented, including whole body wet weight, length, liver wet weight, and testis wet weight. These values were used to calculate BCF, HSI, and GSI. The SSCs were evaluated based on the blind scoring system described by [4,41]. The prominence of nuptial tubercles, dorsal pad and the banding pattern were visually evaluated and scored on a 0–3 scale, where 0 indicates no expression, 1 minor presence, 2 clear presence, and 3 greatest prominence of the respective SSC. A SSC is represented as the sum of individual scores. For the purpose of this analysis, dominant males are considered as those with mean SSC scores of ≥ 2 and subordinate males < 2. Plasma VTG concentrations were measured via a competitive antibody-capture enzyme-linked immunosorbent assays. The VTG concentrations were log-transformed for statistical analyses.

### Data organization

Data points from the studies were organized into tables based on treatment conditions (see S1 Table). Data were tested for the presence of outliers based on Mahalanobis distances (JMP Pro 11 for Macintosh). Outliers (Mahalanobis distance > 3.068) were removed from the subsequent analyses.

### Statistical analyses

Differences in water temperature can have extensive physiological implications for ectothermic vertebrates [42,43]. To assure a high degree of similarity among the environmental conditions evaluated in all studies for which data were available, we tested for similarities in water temperature using one-way ANOVA. Ethanol carrier control data were used for model construction. The distribution profile, a representation of distribution of the single continuous variable using histograms [44], was first run for each variable separately to explore patterns (JMP Pro 11 for Macintosh). The normality test was then performed on SSC and log(VTG) variables to confirm the observed distribution patterns. Since raw data from all studies were available for analyses, weighted averages, a common approach of meta-analysis when original raw data are not available, were not considered necessary. The use of raw data strengthens the overall power of an analysis [33]. Next, a principle component analysis (PCA) was conducted on all continuous variables to reduce data dimensionality and minimize information loss (JMP Pro 11 for Mac software) [45,46]. Given the number of biological variables measured, PCA provides a systematic approach reduce the data dimensions and avoid co-linearity while retaining most of the variability in the data matrix. PCA converts all quantitative variables into uncorrelated weighted linear combinations or principal components (PCs) to reduce the data dimensions by using the most representative PCs. PCA on correlation matrix instead of covariance matrix was used for this study because not all variables are of the same scale and/or shape. The number of retained principle components (PCs) was determined based on the following criteria: eigenvalues > 1 and/or overall variation explained > 80% [45]. Five PCs were chosen for K-Means clustering to assess the natural separation of data points (JMP Pro 11 for Mac software) [46,47]. K-Means clustering technique is useful when working with large data sets. The optimal number of clusters is represented by the cubic cluster criterion (CCC) value; the more positive the CCC, the greater the cluster separation. The number of clusters to retain was determined based on either highest positive or lowest negative CCC. A MANOVA was chosen to evaluate the relationship between clusters and the variables of interest as there were more than one of each dependent and independent variable present in the data matrix. To evaluate the obtained clusters, the MANOVA was run as a follow-up test, where retained PCs (Y1, Y2, Y3, Y4, Y5) represent independent variables, and PC-based clusters the dependent variables (X1 + X2) (JMP Pro 11 for Mac software) [48]. A MANOVA was first run on clusters and PCs, then on clusters and the variables of interest: SSC and log(VTG). The same steps were then repeated for the other three treatments: E1, E2 and SSRIs. For all analyses significance level was set at α = 0.05.

## Results

The current study represents a meta-analysis of multiple studies using a similar experimental design to examine whether intrinsic individual variability, resultant from social status within an experimental treatment condition, needs to be accounted for in toxicological studies. To accomplish this objective, we first tested whether all studies were conducted under comparable environmental study conditions, especially focusing on temperature, a driver of organismal metabolism in ectothermic species. We then examined the social hierarchy, using SSC expression as proxy, for four conditions: control fish from various exposure experiments not exposed to any compounds, fish exposed E1, E2, or various SSRIs. For each condition, we first used a PCA to reduce data dimensionality followed by a K-Means cluster analysis to determine the optimum number of clusters. Finally, a MANOVA was used as a follow-up to the K-Means clustering to identify clusters significantly different from each other and relative to SSC and logVTG variables.

**Fig 2:**
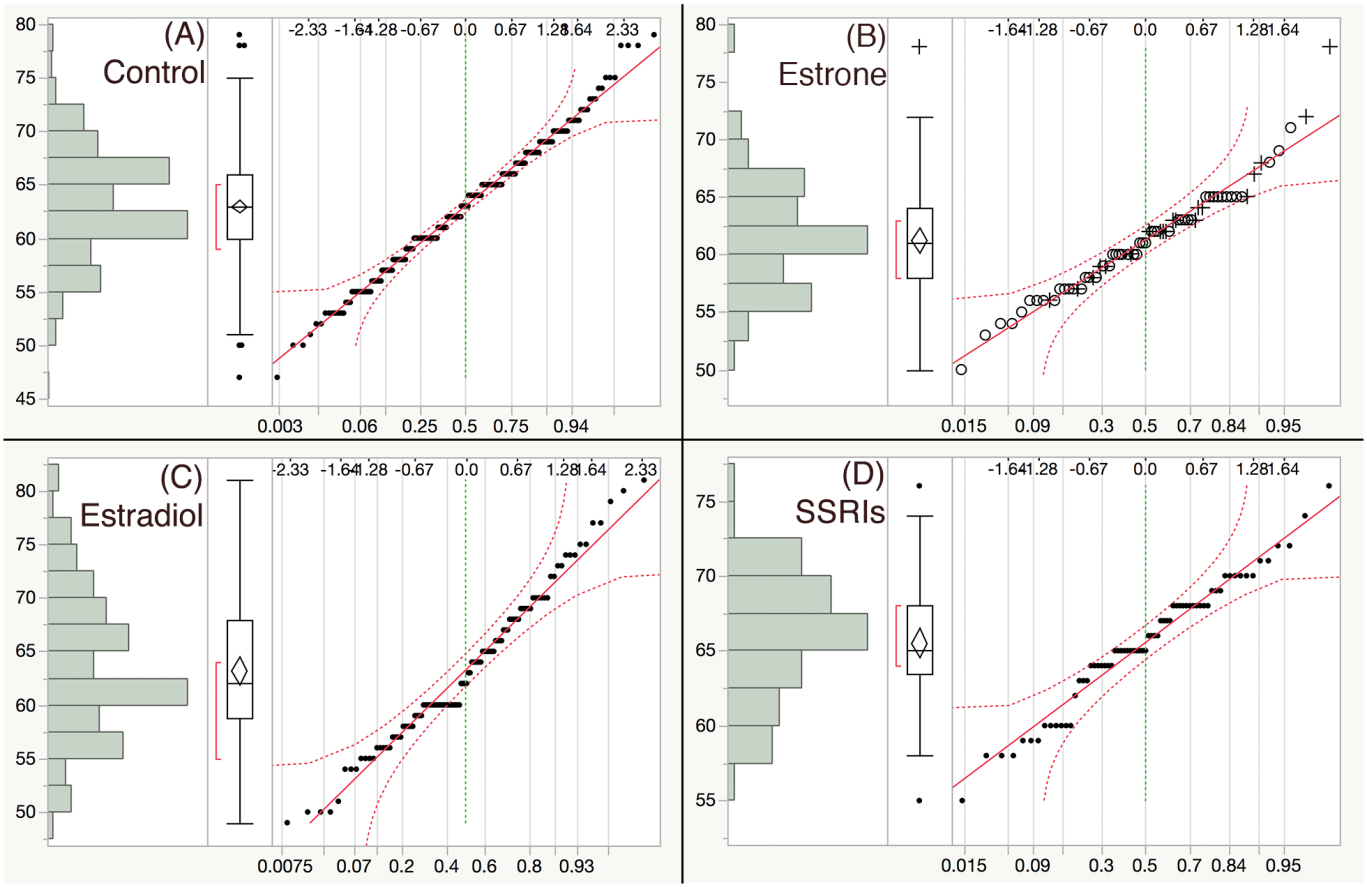
Length distribution of fish. Results of the distribution pattern of “length” variable followed by normality quantile plot from four exposure treatment groups: (A) Control, (B) E1, (C) E2 and (D) SSRIs.

**Fig 3:**
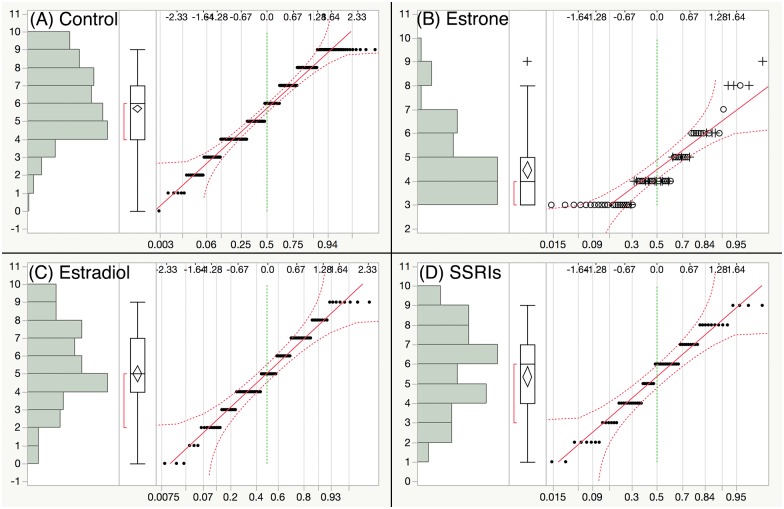
Distribution of secondary sex characteristics. Results of the distribution pattern of “SSC” variable followed by normality quantile plot from four exposure treatment groups: (A) Control, (B) E1, (C) E2 and (D) SSRIs.

**Fig 4:**
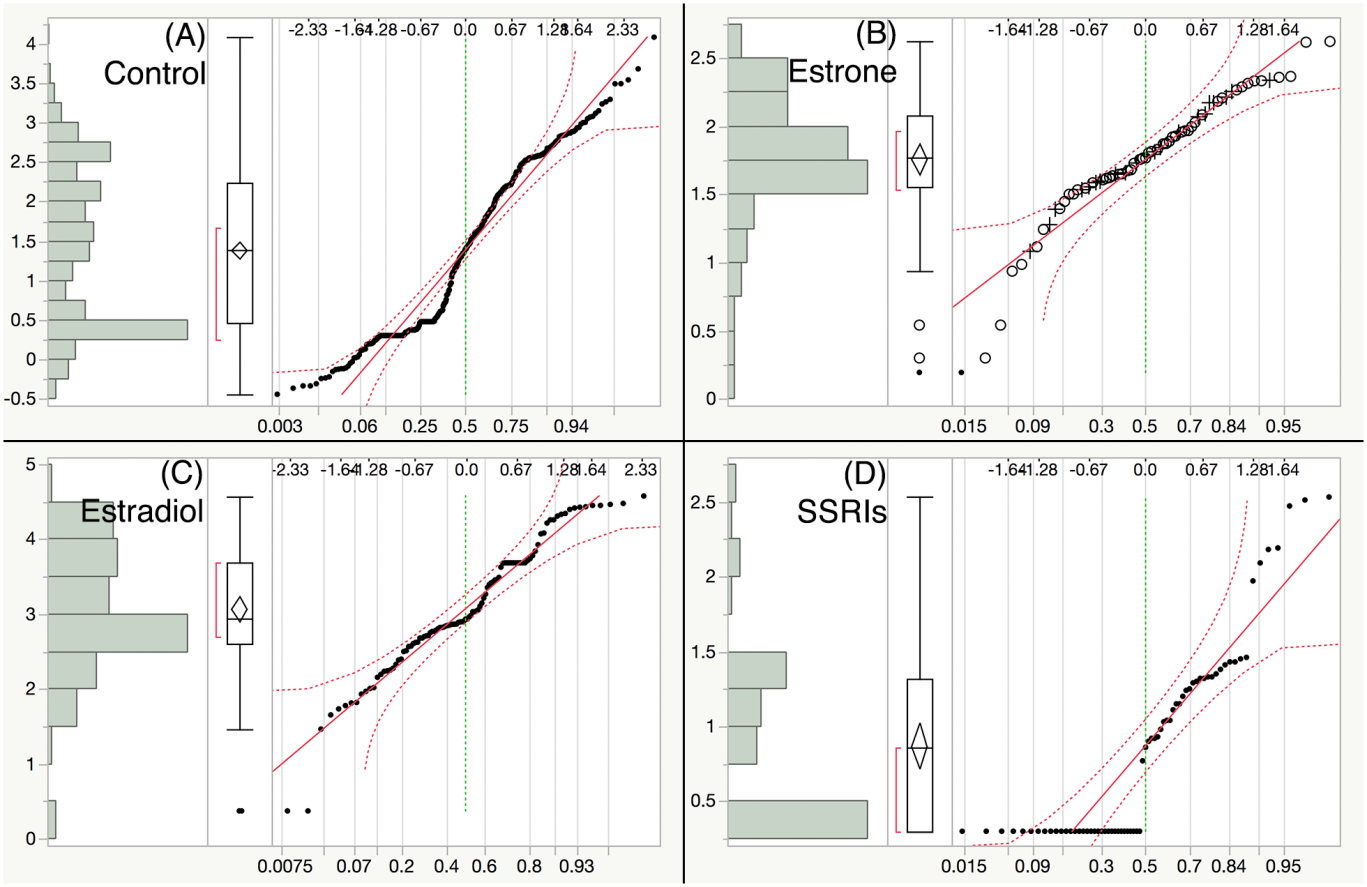
Distribution of vitellogenin across treatments. Results of the distribution pattern of “logVTG” variable followed by normality quantile plot from four exposure treatment groups: (A) Control, (B) E1, (C) E2 and (D) SSRIs.

One-way ANOVA of water temperature indicated that [36] was significantly different from the other studies (S1 Fig). Consequently, this data set was removed from further analysis. The remaining studies were within a 2°C temperature range (S1 Fig) and were included in the subsequent analysis. Following this exclusion, the sample data distributions for all variables were examined. While most variables were normally distributed, logVTG resembled a bimodal distribution. These results were comparable between all treatments, and confirmed with a normality test (Figs 2–4).

### Social hierarchy under control conditions

**Fig 5:**
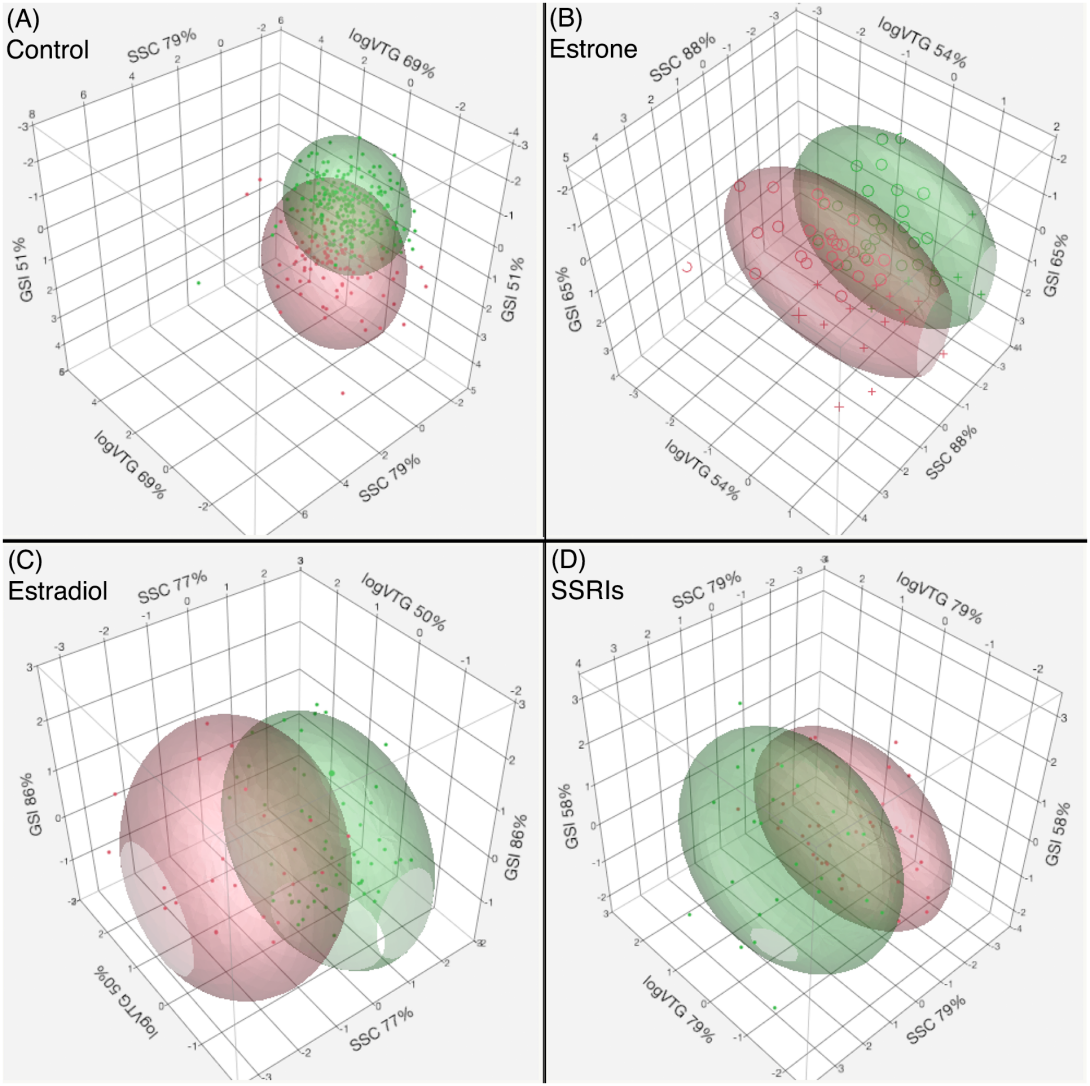
Data separation. Naturally occurring data separation from single sex male exposures was observed with an aid of K-Means cluster analysis performed following PCA. Five PCs were retained based on the number of eigenvalues >1 and minimum percentage of variability explained by these PCs and used for clustering. The most representative variables with contribution percentage driving the PCs are shown on 3D biplots. The treatment groups are: (A) Control, (B) E1, (C) E2 and (D) SSRI.

The PCA yielded five PCs (first five eigenvalues > 1, which together explain 81% of variability). The K-Means cluster analysis run on five PCs yielded two clusters (CCC = -3.2424) as an optimal solution, which supports our hypothesis of the presence of more than one distinct population of male fish within a treatment group (Fig 5A). A MANOVA of the two clusters (dependent variables) versus five PCs (independent variables) showed that clusters are significantly different from each other while already accounting for 81% of variability (p-value < 0.0001). Another MANOVA output resulted in two clusters being significantly different relative to SSC and logVTG variables (p-value < 0.0001). The cluster separation relative to SSC was from 5.71 to 5.76, whereas that relative to logVTG ranged from 0.86 to 2.14 (Fig 5A).

### Social hierarchy under E1 treatment

As with the control condition, five PCs were retained for the E1 treatment (five eigenvalues > 1 and 85% of variability explained). The retention of five PCs again resulted in data separation into two groups (Fig 5B). While two clusters were significantly different from each other (p-value < 0.0001, MANOVA), clusters were not significantly different relative to SSC and logVTG variables (p-value = 0.1748). The mean SSC values decreased to 4.33 and 4.86 relative to those of control, whereas logVTG values remained within 1.76 and 1.81 range (Fig 5B). We observed an increase in VTG synthesis, but to a lesser degree as compared to the E2 treatment.

### Social hierarchy under E2 treatment

Similar to the other treatments, five PCs were retained for the E2 treatment (5^th^ eigenvalue = 0.8924 and 87% of variation is explained). The inclusion of five PCs into a K-Means cluster analysis yielded two distinct populations, similar to the E1 treatment (Fig 5C). A MANOVA on two clusters versus five PCs showed that clusters were significantly different (p-value < 0.0264) (Fig 5C). However, they were again not significantly different relative to SSC and logVTG variables (p-value = 0.1236). The cluster mean SSC values decreased to 4.8 and 5.37 respectively relative to the control, while logVTG values increased to 3.15 and 2.88.

### Social hierarchy under SSRI treatment

Five PCs from the SSRI treatment explained 87% of variation while fifth eigenvalue was at 0.92. Two clusters were identified from clustering of retained PCs (Fig 5D). Clusters by PCs (p-value < 0.0001) and clusters by SSC and logVTG were significantly different according to MANOVA (p-value = 0.0009) (Fig 5D). The separation of cluster mean SSC increased to 5.21 and 6.5 relative to those of control, while logVTG values of 0.70 and 2.17 remained within the range of the Control treatment.

## Discussion

The objective of the current study was to investigate the presence of social hierarchies in single sex fish populations used in toxicological studies and determine how social status modulates the expression of biomarkers following exposure to CECs. Multivariate statistical methods were utilized to investigate whether fish would fall into distinct subpopulations based on their social status, and whether the status influences the response to CEC exposure by modulating VTG synthesis. The original data were extracted from eight independent studies, all of which utilized fish from the same breeding population and were conducted at the same exposure laboratory. The results of this study suggest that under normal laboratory conditions, an initially randomly distributed population of male fish will quickly establish a social hierarchy, as indicated by variations in SSC expression. It is noteworthy that male exposures to exogenous estrogenic compounds, such as E1 and E2, mute male social status towards the subordinate spectrum by suppressing the expression of the SSCs and inducing the production of VTG. The results of male exposures to mood-altering drugs, in contrast, exaggerate social group separation while not inducing VTG biosynthesis beyond what is observed in the control group. The regulations of HPG/HPA axes are suggested as underlying mechanisms for the observed effects (Fig 1). These findings are of consequence to toxicological studies as they suggest that studies using CECs with varying modes of action may differentially affect statistical outcomes of biomarker analyses.

### Impact of estrogenic exposure on social hierarchy

Based on the findings from the cluster analysis and MANOVA, exposure to estrogenic compounds E1 and E2 reduced the variability within the male fathead minnow population by constraining the socially distinct groups to a tighter range relative to the control group, as evidenced by SSC cluster means of 4.33 and 4.86 for E1 treatment; and 4.8 and 5.37 for E2 treatment. In other words, the dominance in male fish was suppressed, as indicated by the expression of SSC, shifting males towards the subordinate spectrum. Such a linkage between estrogenic exposures and social status changes may involve a variety of molecular pathways with complex interactions and regulatory controls (Fig 1). Estrogens could initiate their effects through interactions with estrogen receptors (ERs) in the nucleus [49] or through alternative signaling pathways involving a rapid, non-genomic route initiated by membrane-bound ERs [50]. For example, transcriptional activation of the ER gene at low estrogen concentrations produce more ERs, which initiate the transcription of the VTG gene [51]. The primary location of ER up-regulation upon exposure to exogenous estrogenic compounds is the liver, where VTG synthesis takes place [29,52]. Consequently, increased VTG biosynthesis is correlated with reduced SSCs (Fig 1).

The constraint of social hierarchy among males exposed to both E1 and E2 can be, in part, explained by previous observations that male fish exposed to exogenous estrogens suppress 11-Ketotestosterone production, a primary androgen in teleost fish associated with dominance [23,28]. It is plausible that the reduction in androgen synthesis in male fish results in the downgrading of social status [23,28].

Alternatively, the shift towards subordinance may involve perturbations in the HPG axis. It is known that estrogens modulate the activity of the HPG axis via positive/negative feedback [9,53], which continuously informs the brain of the physiological status of distantly located organs [54]. Exposure to exogenous estrogenic compounds interferes with the stimulatory effects of γ-aminobutyric acid (GABA) that either acts on the hypothalamus to induce the release of GnRH [55], or acts directly on the pituitary gland to induce the release of luteinizing hormone (LH) and follicle stimulating hormone (FSH) [6,54]. Previous studies showed that high levels of estrogens, but not testosterone [56], block the stimulatory effect of GABA and ultimately lead to a reduction of androgens for males [52]. In addition, estrogens inhibit the expression of the *ar* gene that codes for androgen receptors in testis [57], leading to the reduced production of male sex steroids [28]. The reduction in male sex hormone production in either case affects the reproductive status of males [29,52], as manifested in the forms of suppressed aggressive behavior, impaired ability to acquire a nest site under competitive pressure [30], and less prominent SSCs [9]; all consistent with observations in the current study.

### Impact of SSRI exposure on social hierarchy

In contrast to the constraining effects of estrogenic exposure, the exposure to SSRIs relaxed social hierarchy within the male fish population by pushing two socially distinct groups of males further apart (Fig 5C) as evidenced by SSC cluster means of 5.2 and 6.5 for subordinate and dominant groups, respectively. It has previously been established that social stress from aggressive interactions is expressed differently in the brain regions of dominant and subordinate males [17]. The effects of SSRIs are propagated through their complex interactions with serotonin, which interacts with the HPG and HPA axes [18,20,21]. The HPA axis is involved in mediating stressful conditions while altering the serotonin system that is conserved among vertebrates [20]. Studies on teleost fish have also shown an increase in serotonin activity in socially subordinate individuals while inducing the release of cortisol [20,21]. Persistently high levels of cortisol are associated with reduced abilities of an individual to access food, leading to decreased body mass and condition factor, and a chronically activated HPA axis which will further raise cortisol level [7]. Thus chronic exposure to SSRIs elevates serotonin concentrations in the synaptic cleft and prolongs the effects of serotonin, which mimics either a stressful event or a social subordination.

Serotonin, associated with the display of aggressive behavior [17] in male fish, also influences the critical stages of their reproductive development [18]. It has a close association with the HPG axis by inducing LH and FSH release upon stimulation of the pituitary gland by serotonin [17]. Thus, exposures to a SSRI can indirectly affect spermatogenesis in fish by stimulating the release of LH from the pituitary gland [53] through serotonin_2_ receptor [19]. Although serotonin has a stimulatory effect on LH hormones in vertebrates that in turn influence the production of testosterone and consequently spermatogenesis [18], it shows mixed effects between vertebrates on intraspecific aggression. Serotonin exhibits an inverse relationship with aggression in some lizard species [58] whereas studies on invertebrates, crustaceans, resulted in an increase in aggression (Pyle, pers. com.). Johnson et al. [59] confirmed that the serotonin binding receptor mediates aggressive behavior. These authors found that 5-HT_2_ receptor activation reduces the aggression in male fish [59], the same receptor that induces the release of LH from the pituitary gland [6,18]. In contrast, the binding of serotonin to the 5-HT_1A_ receptor induced aggression [59]. Moreover, the increased metabolism and release of serotonin in teleost fish typically occur in response to social stress, the degrees of which differ in extent and timing between the individuals of different social statuses [17,22].

### Consequences of social hierarchy for toxicological studies

The establishment of socially complex communities has been documented for many fishes, including the fathead minnow [3], and is driven by changes in the HPG axis. The dominance of males is determined by the concentration of circulating male sex steroids that ultimately lead to enhanced SSC expression [9,60]. This is consistent with observations that some fish became dominant while others remained subordinate in all of the pooled studies, as indicated by the varying expression of SSCs. It is important to note that, according to our current knowledge, social status is not genetically determined, but rather regulated at the level of the endocrine state of a male. The genetic contributions to social status, if any, remain to be identified. Thus, dominant males are not limited to those genetically predisposed to achieve the highest level in a social organization [61].

Toxicologists designing exposure experiments with single-sex test organisms should carefully evaluate the implications of social hierarchy. In experiments in which exposure compounds are likely to homogenize the population by suppressing dominance, social hierarchy may have little effect on the statistical power of the study design. This appears to be the case for the estrogenic CEC assessed in the current study and likely for other CECs that reduce the overall social hierarchy. In contrast, studies examining the effects of CECs that liberate social constraints, such as the SSRIs examined in the current study, may undermine the statistical power of the experimental design. If possible within the constraints of the hypothesis being tested, experiments that separate individuals or create breeding pairs may be advantageous to single-sex exposure experiments as they avoid the development of a social hierarchy during the experimentation. However, tests subjects at the onset of the study may already represent varying dominance based on their prior housing in larger breeding groups, a common holding approach in toxicological testing facilities. In those instances, recording of the social status at the beginning of each experiment and equal distribution of dominant and subordinate test subjects among treatments may reduce the effect of existing social hierarchy. The current study focused on males of a species exhibiting sexual dimorphism that is known to establish social hierarchies [3,4]. However, female fish exposed to androgenic CECs may also develop social hierarchies as some females become masculinized. It is therefore prudent to assess any experimental design using hierarchy-forming species in light of the impacts of social hierarchies.

In summary, we demonstrated in the current study that male fathead minnows separate into physiologically distinct subpopulations within treatment groups. We showed that while social hierarchy is present in all social animals, the dominance of individuals can be affected depending on their initial status and the class of contaminants they are exposed to. These findings suggest that treating a fish population as homogeneous may lead to an increased chance of Type II error (false negative), which implies that the effects of some endocrine-disrupting compounds may have been underestimated.

## Supporting information

S1 Fig Temperature profile of pooled studies. Pooled studies were compared based on the temperatures, at which fish were reared during the exposure experiments. One-way Anova with all pairs Tukey-Kramer post-test (α = 0.05) were conducted to investigate the differences between the studies.
(TIFF)

S1 Table Raw data file. All data used in the current manuscript.
(XLSX)
