# Supplementary figures and images for: Social hierarchy modulates responses of fish exposed to contaminants of emerging concern
Source: PLoS One. 2017 Oct 19;12(10):e0186807. doi: 10.1371/journal.pone.0186807 (PMC5648243; doi:10.1371/journal.pone.0186807)

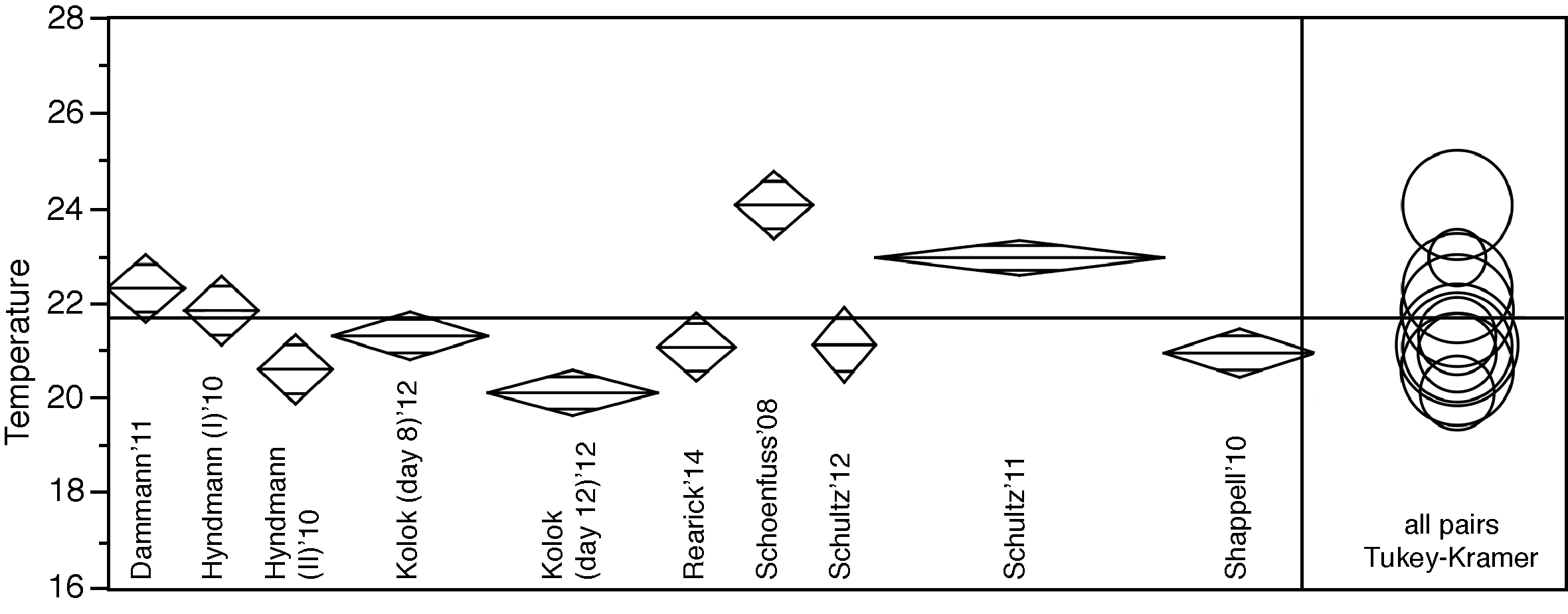

Supplement: S1 Fig — Pooled studies were compared based on the temperatures, at which fish were reared during the exposure experiments. One-way Anova with all pairs Tukey-Kramer post-test (α = 0.05) were conducted to investigate the differences between the studies. (TIFF) [file pone.0186807.s001.tiff]
